# CMZ Reversed Chronic Ethanol-Induced Disturbance of PPAR-α Possibly by Suppressing Oxidative Stress and PGC-1α Acetylation, and Activating the MAPK and GSK3β Pathway

**DOI:** 10.1371/journal.pone.0098658

**Published:** 2014-06-03

**Authors:** Tao Zeng, Cui-Li Zhang, Fu-Yong Song, Xiu-Lan Zhao, Ke-Qin Xie

**Affiliations:** Institute of Toxicology, School of Public Health, Shandong University, Jinan City, Shandong Province, People's Republic of China; Florida International University, United States of America

## Abstract

**Background:**

Cytochrome P4502E1 (CYP2E1) has been suggested to play critical roles in the pathogenesis of alcoholic fatty liver (AFL), but the underlying mechanisms remains unclear. The current study was designed to evaluate whether CYP2E1 suppression by chlormethiazole (CMZ) could suppress AFL in mice, and to explore the underlying mechanisms.

**Methods:**

Mice were treated with or without CMZ (50 mg/kg bw, i.p.) and subjected to liquid diet with or without ethanol (5%, w/v) for 4 weeks. Biochemical parameters were measured using commercial kits. The protein and mRNA levels were detected by western blot and qPCR, respectively. Histopathology and immunohistochemical assay were performed with routine methods.

**Results:**

CYP2E1 inhibition by CMZ completely blocked AFL in mice, shown as the decline of the hepatic and serum triglyceride levels, and the fewer fat droplets in the liver sections. Chronic ethanol exposure led to significant decrease of the mRNA and protein levels of peroxisome proliferator-activated receptor α (PPAR-α), which was blocked by CMZ co-treatment. CMZ co-treatment suppressed ethanol-induced oxidative stress, overproduction of tumor necrosis α (TNF-α), and decrease of protein levels of the PPAR-α co-activators including p300 and deacetylated PGC1-α. Furthermore, CMZ co-treatment led to the activation of AMP-activated protein kinase (AMPK), mitogen-activated protein kinase (MAPK), and PI3K/Akt/GSK3β pathway. However, chronic ethanol-induced decline of acyl-CoA carboxylase (ACC) and fatty acid synthase (FAS) protein levels was partially restored by CMZ, while the activation of autophagy appeared to be suppressed by CMZ.

**Conclusion:**

These results suggested that CMZ suppressed chronic ethanol-induced oxidative stress, TNF-α overproduction, decline of p300 protein level and deacetylation of PGC1-α, and activated AMPK, MAPK, and PI3K/Akt/GSK3β pathway, which might contribute to the activation of PPAR-α and account for the protection of CMZ against AFL.

## Introduction

Alcoholic liver disease (ALD) remains to be one of the most common etiologies of liver diseases and is a major cause of morbidity and mortality worldwide [1]. ALD is a pathological process characterized by progressive liver damage from steatosis to steatohepatitis, fibrosis and finally cirrhosis [2]. Alcoholic fatty liver (AFL) is the earliest and most common pathological form of ALD. Although AFL was considered benign in the past, recent studies have demonstrated that fat accumulation renders the liver more vulnerable to toxins such as endotoxin, and AFL is easily progressed to hepatitis, fibrosis and even cirrhosis [3]. It was reported that AFL developed in about 90% of individuals who drank more than 60 g/day of alcohol, but might also occur in individuals who drank less [4], [5]. Other studies suggested that progression to fibrosis and cirrhosis occurred in 5%–15% of AFL patients despite abstinence [6], [7]. Thus, AFL should be the optimal intervene phase for the prevention of ALD to more serious phases such as fibrosis and cirrhosis [8].

The mechanisms for AFL have been extensively studied in the past decades, and several important factors including peroxisome proliferator-activated receptor α (PPAR-α), sterol regulatory element-binding protein-1c (SREBP-1c), AMP-activated protein kinase (AMPK), autophagy, cytochrome P4502E1 (CYP2E1), and cytokines such as adiponectin and tumor necrosis α (TNF-α) have all been proposed to be involved in the development of AFL [2], [9]–[11]. Among these factors, CYP2E1 has gained great attention because it could mediate the metabolism of ethanol, to generate reactive oxygen species (ROS) and to be induced by ethanol [12]. CYP2E1 is a member of the cytochrome P450 superfamily, which is a group of heme-containing proteins with multiple functions including the metabolism of xenobiotics including drugs, toxins, carcinogens, and endogenous substrates [13]. CYP2E1 could catalyze the two electron oxidation of ethanol to acetaldehyde, and can also promote the one electron oxidation to produce 1-hydroxyethyl radicals [14]. Recently, Lu *et al.* demonstrated that CYP2E1 was a pivotal contributor to AFL by using CYP2E1 knockdown mice [15]. In that study, 4 weeks of ethanol-containing Lieber-DeCarli liquid diet treatment induced significant microvesicular fatty liver in wild type mice, but not in the CYP2E1-deficient mice [15]. However, the potential links between CYP2E1 and AFL remain to be elucidated. Furthermore, there were still some studies which denied the critical roles of CYP2E1 in the etiology of ALD. For example, in the study by Wan *et al.*, the authors found that PPAR-α was inhibited by ethanol in wild type mice liver, and the inhibitory effect of ethanol was more prominent in the CYP2E1 deficient mice [16]. Another study showed that CYP2E1 only played a small role in mechanisms of early ALD in mice [17]. Clearly, further studies are needed to elucidate the correlation between CYP2E1 and AFL.

The liver triglyceride (TG) metabolism is mainly controlled by three independent pathways, i.e. PPAR-α-regulated fatty acid oxidation pathway, SREBP-1c-regulated hepatic fatty acid synthesis, and recently reported autophagy-mediated lipid decomposition [18]–[23]. PPAR-α and SREBP-1c are two important nuclear transcription factors, which regulate a battery of genes involved in fatty acid oxidation and fatty acid synthesis, respectively. PPAR-α governs the expression of many genes involved in the fatty acid peroxisomal and mitochondrial β oxidation such as acyl-CoA oxidase, long chain and medium chain acyl-CoA dehydrogenases, carnitine palmitoyl-CoA transferase, and also regulates fatty acid transport protein, liver fatty acid-binding protein, and CYP4A (the enzyme involved in the microsomal fatty acid ω-oxidation). In contrast, SREBP-1c plays an important role in the regulation of transcription of genes involved in hepatic lipogenesis including acyl-CoA carboxylase (ACC) and fatty acid synthase (FAS)[22]. Accumulating evidences have demonstrated that both PPAR-α suppression and SREBP-1c activation induced by ethanol play important roles in the development of AFL [20], [21], [24]–[27]. Recently, autophagy has also been demonstrated to regulate the intracellular lipid, and both acute and chronic ethanol exposure could lead to the activation of autophagy, which may help to clear the damaged mitochondria and accumulated lipid droplet in the liver [28], [29].

In order to further elucidate the roles of CYP2E1 in AFL, and to explore the underlying mechanisms, chlormethiazole (CMZ), a specific CYP2E1 inhibitor, was used to inhibit CYP2E1 in mice orally exposed to 4 weeks of Lieber-DeCarli liquid diet (containing 5% ethanol, w/v). We aimed to address: 1) whether CYP2E1 inhibition by CMZ could suppress chronic ethanol-induced fatty liver? 2) Whether the protective effects of CMZ were associated with PPAR-α pathway? And if so, which are the underlying mechanisms? And 3) whether the protective effects of CMZ were related with SREBP-1c pathway or autophagy.

## Materials and Methods

### Materials

CMZ and primary antibodies against LC3 and p62/SQATM1 were bought from Sigma (St. Louis, MO, USA). Primary antibodies against PPAR-α (H-98), RXR-α (D-20), p300(C-20), PGC-1α (H-300), Sirt-1(H-300), SREBP-1c (H-160), FAS (A-5), GSK-3β (H-76), and phospho-GSK-3β (Ser^9^) were purchased from Santa Cruz (Santa Cruz, CA, USA). Primary antibodies against AMPKα, phospho-AMPKα (Thr^172^), ACC, phospho-ACC (Ser^79^), Erk1/2, phospho-Erk1/2, p38, phospho-p38, JNK, phospho-JNK, Akt, phospho-Akt(Ser^473^), phospho-Akt(Thr^308^), and PI3K-p110α, were provided by Cell Signaling Technology (Beverly, MA, USA). Rabbit polyclonal PI3K-p85 (N-SH2 domain) antibody was obtained from Millipore (Billerica, MA, USA). Rabbit polyclonal DGAT2 antibody was bought from Novus Biologicals (Littleton, CO, USA). Western blotting detecting reagents (ECL kits) was provided by Millipore. Biochemical assay kits of serum alanine aminotransferase (ALT), aspartate aminotransferase (AST), TG, malondialdehyde (MDA) and glutathione (GSH) were supplied by Nanjing Jiancheng Bioengineering Institute (Nanjing, China). Tissue TG assay kit was purchased from Applygen Technologies Inc. (Beijing, China). Mouse TNF-α ELISA Kit and mouse adiponectin/Acrp30 ELISA kit were bought from Millipore and R&D systems (Minneapolis, MN, USA), respectively. Enzychrom ethanol assay kit was provided by Bioassay systems (Hayward, CA, USA). All other reagents were purchased from Sigma unless indicated otherwise.

### Animals and Experimental Design

#### Ethics Statement

The experiments were conducted in accordance with the NIH Guide for Care and Use of Laboratory Animals and the principles in the ‘‘Use of Animals in Toxicology’’, and were approved by the Ethics Committee of Shandong University Institute of Preventive Medicine (Permit Number:20120701).

#### Animals

Specific pathogen-free (SPF) male Kun-Ming mice (18–22 g) were provided by the Laboratory Animal Center of Shandong University (Jinan, China). The mice were maintained in a temperature-controlled environment (20–22°C) with a 12-h light: 12-h dark cycle and 50-55% humidity during the whole experiment. Animal housing and care followed currently accepted standards of the NIH Guide for Care and Use of Laboratory Animals and the principles in the ‘‘Use of Animals in Toxicology’’.

#### Experimental Design

After 3 days of acclimation to the animal facility with the standard chow and tape water, the mice were randomly divided into 4 groups (n = 8), i.e. control group, ethanol group, CMZ group, and CMZ/ethanol group. The mice in ethanol group and CMZ/ethanol group were treated with Lieber-DeCarli liquid diet containing 5% (w/v) ethanol [30]. The ethanol was gradually introduced into diet from 1% to 5% (w/v) over a 1 week period for adaptation. The animals in other two groups received isocaloric liquid diet in which ethanol was replaced by dextrin-maltose. CMZ was administrated to mice in CMZ group and CMZ/ethanol group by intraperitoneal injection with a dose of 50 mg/kg body weight every other day, while animals in other two groups received equal volume of sterile saline. The mice body weight was measured once a week during the feeding period. All the liquid diet were freshly prepared from powder every day, and the dietary intake of control group mice were matched to those of the ethanol-fed mice by pair feeding the same volume of isocaloric liquid diet for 4 weeks. The mice were sacrificed at the end of the experiment. The blood was collected and centrifuged at 1000 g for 10 min to separate the serum. The livers were removed quickly, weighted, and then excised into several fragments. All the samples except for those for pathological examination were stored at −80°C until analysis.

### Biochemical assay

The levels of serum ALT, AST and TG, and hepatic MDA and GSH were measured according to the protocols of commercial assay kits bought from Nanjing Jiancheng Bioengineering Institute (Nanjing, China). The hepatic TG content was detected using the tissue TG assay kit obtained from Applygen Technologies Inc. (Beijing, China). Serum ethanol concentration was determined using the Enzychrom ethanol assay kit provided by Bioassay systems (Hayward, CA, USA).

### Measurement of the serum adiponectin and TNF-α levels

The serum adiponectin and TNF-α levels were measured by use of ELISA kits provided by Millipore (Bedford, MA, USA) and R&D Systems (Minneapolis, MN, USA), respectively.

### Liver pathological examination

Liver pathological examination was performed using 3 methods, i.e. hematoxylin and eosin (H&E) staining, Sudan III staining, and oil red O staining. For the H&E staining, pieces of liver from the same lobes were fixed in 10% formalin for about 24 h. The paraffin sections were prepared and cut into 5 µm thick sections using a rotary microtome (Lieca, Germany). The sections were passed through xylene, alcohol, water, and then stained with H&E dye [31]. For the Sudan III and oil red O staining, frozen sections (about 8 µm) were prepared, affixed to microscope slide, and allowed to air-dry at room temperature. The liver sections were fixed in 10% formalin for 5 min, stained in Sudan III or oil red O dyes, and then counterstained with hematoxylin for 30 seconds [8]. The sections were viewed and the representative photographs were captured at 400× magnifications by using a Nikon microscope (Nikon, Melville, NY, USA) equipped with a Metamorph image acquisition and analysis system (Chester, PA, USA).

### Immunohistochemical staining

Liver frozen sections (5 µm) were prepared, mounted on APEX coated slides, and fixed in cold acetone for 10 min at 4°C. Endogenous peroxidase activity was blocked using 3% H2O2, and 5% normal goat serum was used to block nonspecific staining. After that, sections were incubated with CYP2E1 primary antibody (1∶500, Abcam, UK) at 4°C overnight, and then incubated with poly peroxidase-anti-mouse/rabbit IgG (PV-9000, ZSGB-BIO, China) for 30 min at room temperature. After wash in PBS for 3 times, the reaction was detected by incubating with 3′, 5′-diaminobenzidine (DAB) for 30 seconds at room temperature. Then the sections were washed in tap water and counterstained with hematoxylin for 30 seconds, and the images were viewed and captured at 400× magnifications using light microscope (Nikon, Melville, NY, USA).

### Transmission electron microscopy (TEM)

Fresh liver pieces (about 1 mm^3^ cubes) were fixed in 2% glutaraldehyde overnight at 4°C. The samples were then postfixed with 1% osmium tetroxide (OsO4) solution for 2 h, dehydrated through graded ethanol series. The samples were embedded in epoxy resin and were cut into 60–70 nm thick sections using LKB ultramicrotome. The sections were double-stained with uranyl acetate and lead citrate, and observed using a transmission electron microscope (JEM-2000ex, JEDL Co., Japan).

### Detection of the CYP2E1 activity

CYP2E1 activity was detected as previously reported [32], [33]. Briefly, liver tissue was homogenized in 4 volume ice-cold TMS buffer (50 mM Tris-HCL, 6.4 mM MgCl2, 0.2 M saccharose, pH 7.5). The resulting homogenate was centrifuged at 12, 000 g for 15 min and the supernatants were further centrifuged at 105, 000 g for 60 min. The final pellet was reconstituted in the above buffer and considered as the microsome sample, and used for the detection of CYP2E1 activity with aniline as the substrate.

### Real-time PCR analysis

Total RNA was isolated from mice liver using Trizol reagent (Invitorgen, USA). 2 µg of total RNA was reverse transcribed to cDNA at 42°C for 1 h using Oligo dT-Adaptor primer and RevertAid M-Mul V Transcriptase according to the manufacture's protocol (Fermentas, UK). Quantitative real-time PCR (qPCR) was performed using Roche lightCycler480 SYBR Green I Master (Roche, Germany) to quantify the mRNA levels of PPAR-α, SREBP-1, RXR-α, PGC-1α, ACC, and FAS. Glyceraldehyde 3-phosphate dehydrogenase (GAPDH) was used as an internal loading control. The primers were synthesized by Sangon Biotech Co., Ltd (Shanghai, China), and were listed in **Table 1**. The PCR amplification reactions were performed using Roche LightCycler 480 Instrument (Roche, Germany).

**Table 1:** Gene-specific primers used in quantitative real-time PCR.

Gene	GeneBank ACC.No.	Forward primer (5′-3′)	Reverse primer (5′-3′)
PPAR-α	NM_001113418	TGGCAAAAGGCAAGGAGAAG	CCCTCTACATAGAACTGCAAGGTTT
SREBP-1	NM_011480	GATGTGCGAACTGGACACAG	CATAGGGGGCGTCAAACAG
PGC-1α	NM_001127330	CCCTGCCATTGTTAAGAC	GCTGCTGTTCCTGTTTTC
FAS	NM_008904	GGAGGTGGTGATAGCCGGTAT	TGGGTAATCCATAGAGCCCAG
ACC-α	NM_133360	CTGGCTGCATCCATTATGTCA	TGGTAGACTGCCCGTGTGAA
RXR-α	NM_011305	CCCCTTCTTGGTGATTTGAACA	GCACCACAATGTCCCAGTGA
GAPDH	NM_001001304	GCATGGCCTTCCGTGTTCC	GGGTGGTCCAGGGTTTCTTACTC

### Western blot analysis

The total protein extracts were prepared using RIPA buffer (50 mM Tris, 150 mM NaCl, 1% Triton X-100, 1% sodium deoxycholate, 0.1% sodium dodecyl sulphate (SDS), 1 mM phenylmethylsulfonyl fluoride (PMSF), 1 mM Na3VO4, 5 mM NaF, and 1% cocktail protein protease inhibitors (Sigma), pH 8.0) as we previously reported [31], [34]. Protein samples were mixed with 3× loading buffer, and heated at 100°C for 5 min. The pretreated protein samples (about 20-50 µg) were separated by electrophoresis in an 6%–15% denatured polyacrylamide gel, transferred to a polyvinylidene fluoride (PVDF) membrane (Immobilon-P; Millipore Corp., Bedford, MA, USA). The membranes were blocked with 5% nonfat milk solution for 1 h at room temperature, and then incubated with specific primary antibodies overnight at 4°C. After washes in TBST for 3 times with 10 min each, the membranes were incubated with horseradish peroxidase (HRP)-conjugated anti-rabbit or anti-mouse antibodies for 1.5 h at room temperature. The membranes were then washed for 3 times with 10 min each in TBST, and proteins were visualized using an enhanced chemiluminescence (ECL) western blotting detection reagent. The immunoreactive bands of proteins were scanned by using Agfa Duoscan T1200 scanner, and the digitized data were quantified as integrated optical density (IOD) using Kodak Imaging Program. To ensure equal loading, the results were normalized by β-actin.

### PGC-1α acetylation assays

PGC-1α acetylation was detected by immunoprecipitation [35]. In brief, PGC-1α protein was immunoprecipitated with liver protein extracts by an anti-PGC-1α antibody (Santa Cruz), and then the PGC-1α levels and acetylation were detected using specific antibodies for PGC-1α (Santa Cruz) and acetyl lysine (Cell Signaling), respectively.

### Statistical analyses

All data were expressed as mean and standard deviation (SD). SPSS13.0 statistical software was used for statistical analysis. The data were analyzed using one-way analysis of variance (ANOVA) followed by LSD's post hoc tests. Differences were considered statistically significant at *p*<0.05

## Results

### CMZ effectively inhibited ethanol-induced activation of CYP2E1 in mice liver

**Figure 1:**
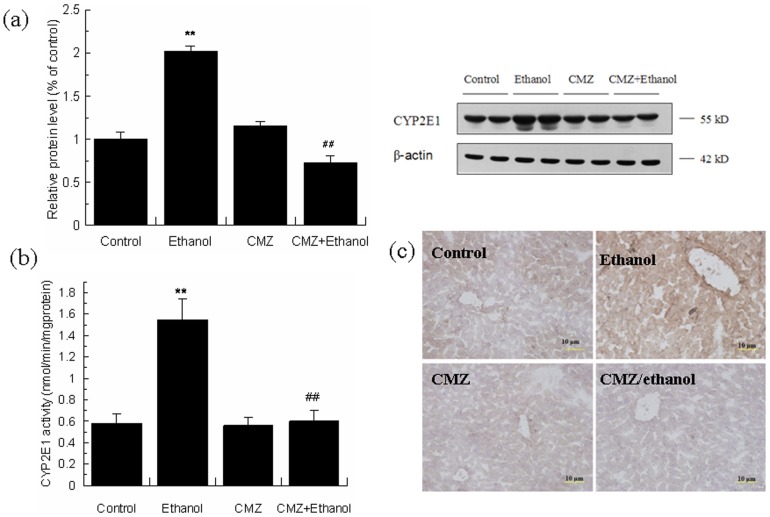
CMZ prevented ethanol-induced activation of CYP2E1 in mice liver. (a) Protein levels of CYP2E1 were detected by western blot; (b) CYP2E1 activity; (c) Immunohistochemical staining of CYP2E1. Data were presented as mean ± SD. **P<0.01, compared with control group; ^##^ *P<*0.01, compared with ethanol group.

Chronic ethanol exposure led to significant activation of CYP2E1, shown by the increase of the protein level and activity of CYP2E1. CMZ co-treatment effectively inhibited chronic ethanol-induced CYP2E1 activation, and restored the protein level and activity of CYP2E1 to the levels of the control group mice (**Fig.1a & 1b**). Immunohistochemical study showed weak staining of CYP2E1 protein in the perivenular region of liver sections of mice in control, CMZ and CMZ/ethanol groups, while greatly increased staining was observed in the liver sections of ethanol group mice (**Fig.1c**).

### CYP2E1 inhibition by CMZ blocked chronic ethanol-induced fatty liver in mice

**Figure 2:**
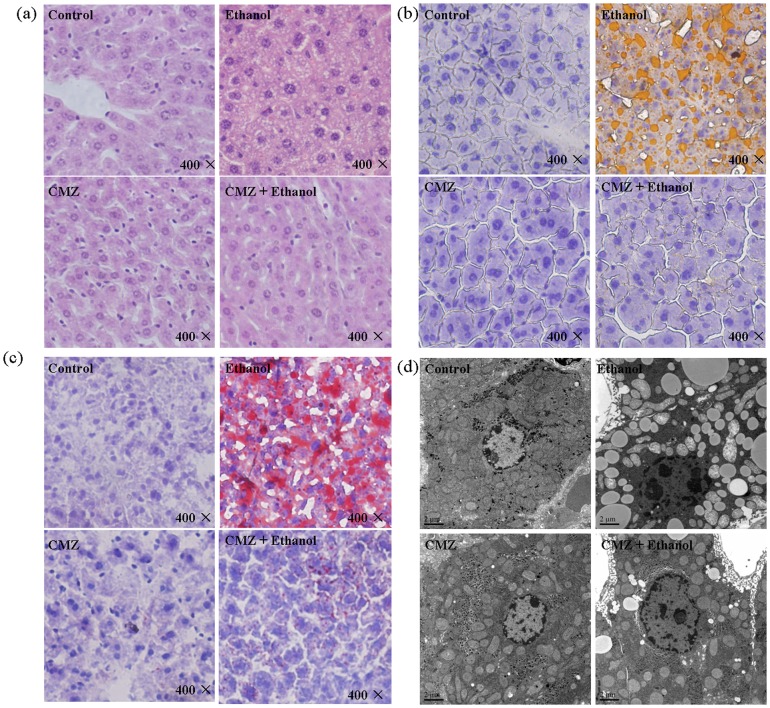
CYP2E1 inhibition by CMZ completely blocked chronic ethanol-induced lipid accumulation in mice liver. Mice were treated with liquid diet with or without ethanol in the presence/absence of CMZ for 4 weeks. (a) H&E staining; (b) Sudan III staining; (c) Oil red O staining; (d) Ultrastructural examination.

As shown in **Table 2**, significant increase of the serum TG level, hepatic TG level, liver index, and ALT and AST activities were observed in ethanol group mice. However, CMZ co-treatment suppressed the increase of the above parameters except the liver index (the liver weight/body weight ×100%), which indicated the amelioration of ethanol-induced liver damage by CMZ. Liver pathological examination was also performed to investigate hepatic fat accumulation in mice liver (**Fig. 2**). H&E staining showed massive microvesicular steatosis in ethanol group mice liver, which was obviously alleviative in CMZ/ethanol group mice liver (**Fig. 2a**). The results of the fat specific staining, Sudan III and oil red O staining, showed massive yellow- or red-stained lipid droplets in liver sections of ethanol group mice, while no obvious fat droplets were presented in the liver sections of CMZ/ethanol group mice (**Fig. 2b&2c**). The ultrastructural examination further demonstrated CYP2E1 inhibition by CMZ suppressed chronic ethanol-induced fat accumulation in mice liver (**Fig. 2d**).

**Table 2:** Effects of CMZ and ethanol on the levels of serum ALT, AST, TG, ethanol, TNF-α, adiponectin, and hepatic TG, MDA, GSH.

	Parameters	Control	Ethanol	CMZ	CMZ + Ethanol
Serum	ALT (U/L)	20.98±4.42	46.19±7.95**	23.51±1.81	28.83±8.07^##^
	AST (U/L)	20.52±4.58	39.89±5.60**	23.78±5.29	19.07±5.67^##^
	TG (mg/mL)	1.88±0.13	2.35±0.46**	1.67±0.28	1.90±0.12^##^
	TNF-α (pg/mL)	11.41±1.67	26.28±3.42**	12.50±1.52	13.28±2.80^##^
	Adiponectin ( µg/mL)	8.34±1.97	7.64±0.79	8.06±1.42	8.21±1.01
	Ethanol (mg/dL)	—	199.8±49.7	—	187.0±46.9
Hepatic	TG ( µmol/g liver)	27.52±4.61	57.17±3.93**	31.64±3.79	21.99±2.72^##^
	MDA (nmol/mg protein)	1.13±0.23	1.75±0.19**	1.09±0.13	1.16±0.17^##^
	GSH (mg/g protein)	1.51±0.20	1.08±0.15**	1.37±0.07	2.02±0.35^##^
	Liver index	3.83±0.29	5.50±0.44**	4.47±0.72	5.51±0.39

Liver index  =  liver weight/body weight ×100%;**P<0.01, compared with Control group mice; ^##^ *P<*0.01, compared with ethanol group mice.

### CMZ suppressed the decrease of protein levels of PPAR-α, p300 and Sirt-1, and inhibited the increased acetylation of PGC-1α induced by ethanol

**Figure 3:**
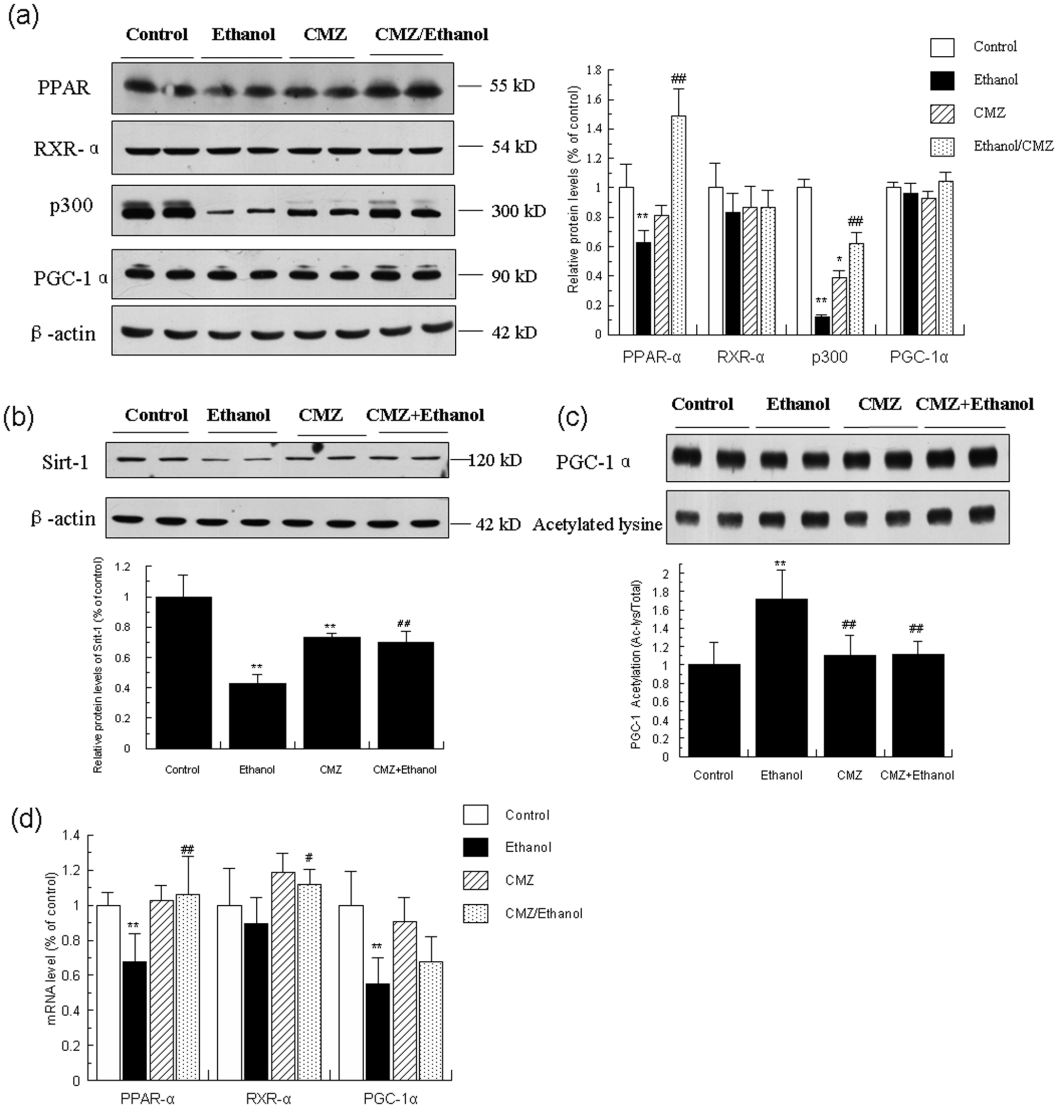
CMZ prevented the decrease of protein levels of PPAR-α, p300 and Sirt-1, and inhibited the increased acetylation of PGC-1α induced by ethanol. Total protein samples were prepared using RIPA buffer, and protein levels of PPAR-α, RXR-α, p300, PGC-1α, and Sirt-1 were detected by western blot. The acetylation of PGC-1α was determined by immunoprecipitation analysis. The mRNA levels of PPAR-α, RXR-α, and PGC-1α were measured using qPCR. (a) Protein levels of PPAR-α, RXR-α, p300, and PGC-1α; (b) Protein levels of Sirt-1; (c) Acetylation of PGC-1α; (d) mRNA levels of PPAR-α, RXR-α, and PGC-1α. Data were presented as mean ± SD from at least 3 independent experiments, and expressed as the percentage of the control. *P<0.05, **P<0.01, compared with control group; ^#^ *P<*0.05, ^##^ *P<*0.01, compared with ethanol group.

Emerging evidence indicates that the nuclear receptor PPAR-α plays a crucial role in the pathogenesis of AFL, due to its important roles in the transcriptional control of the expression of many enzymes involved in intracellular transport and oxidation of fatty acid [10], [22]. Therefore, we investigated the protein and mRNA levels of PPAR-α in mice liver by western blot and qPCR, respectively. As shown in **Fig.3**, the protein level and mRNA level of PPAR-α in the liver of ethanol group mice were significantly decreased when compared with the control group mice, while CMZ co-treatment led to a significant increase of the PPAR-α protein and mRNA levels in mice liver.

In the nucleus, PPAR-α exist as heterodimers with retinoid X receptor (RXR) - bound to DNA with corepressor molecules. Upon ligand activation, PPAR-α undergoes conformational changes that facilitate the dissociation of co-repressor molecules and invoke a spatiotemporally orchestrated recruitment of transcription cofactors including coactivators and coactivator-associated proteins [36]. We then detected whether ethanol and/or CMZ could affect the RXR-α and PPAR-α related coactivators including p300 and PPAR-γ coactivator-1α (PGC-1α). As shown in **Fig.3**, the protein levels of RXR-α and PGC-1α were not significantly affected by ethanol and CMZ, while the decline of the mRNA levels of RXR-α and PGC-1α induced by ethanol was partially suppressed by CMZ co-treatment. However, the protein level of p300 was dramatically decreased in ethanol group mice liver compared with that of control group mice, which was significantly inhibited by CMZ co-treatment. Chronic ethanol exposure also led to the increased acetylation of PGC-1α, which was restored to the normal value by CMZ co-treatment (**Fig. 3c**). The protein level of Sirt-1, a NAD^+^-dependent protein deacetylase, was significantly decreased in the liver of ethanol group mice, which was also significantly inhibited by CMZ co-treatment (**Fig. 3b**).

### CMZ co-treatment suppressed ethanol-induced oxidative stress and the elevation of the serum TNF-α

CYP2E1 is a major contributor to ethanol-induced oxidative stress[37], [38], and ethanol-induced oxidative stress could lead to the overproduction of TNF-α [39], which will then down-regulated the expression of PPAR-α [40]. We then investigated the biomarkers for oxidative stress and serum levels of TNF-α. As shown in **Table 2**, compared with those of control group mice, significant increase of the hepatic MDA level (a biomarker for oxidative stress), decrease of GSH, and increased TNF-α level were observed in ethanol group mice. CMZ co-treatment significantly suppressed the elevation of hepatic MDA level, dramatically increased the hepatic GSH level, and inhibited the serum TNF-α level. We also detected another cytokine, adiponectin, which has also reported to regulate the activity of PPAR-α [41], but did not find significant changes between each groups.

### CMZ co-treatment enhanced the phosphorylation and activation of AMPK

**Figure 4:**
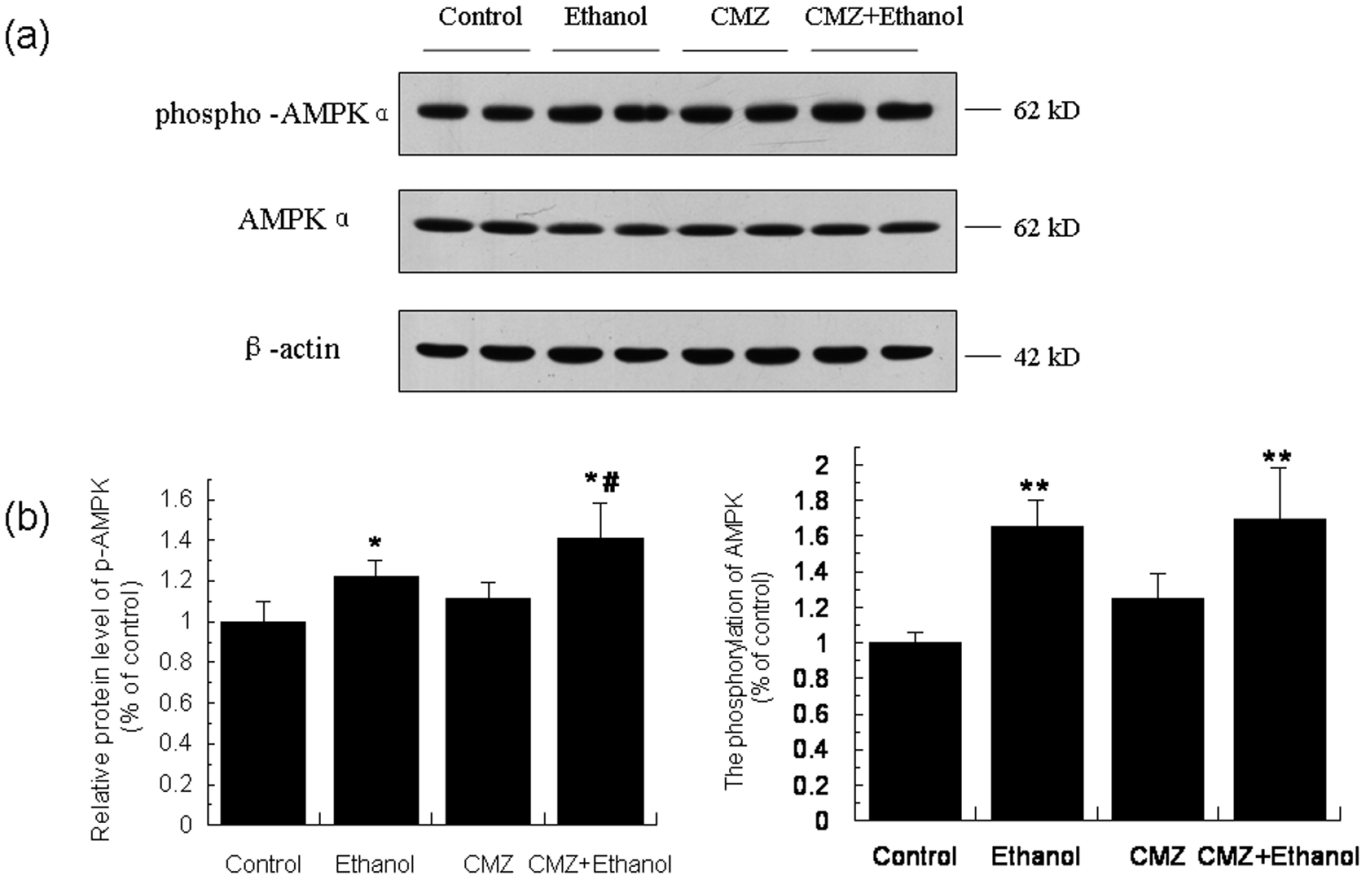
The phosphorylation of AMPK was increased in the liver of CMZ/ethanol group mice. (a) Representative western blot bands for phosphor-AMPK and AMPK; (b) Quantitative data analyses. Data were presented as mean ± SD from at least 3 independent experiments, and expressed as the percentage of the control. *P<0.05, **P<0.01, compared with control group; ^#^ *P<*0.05, compared with ethanol group.

AMPK is a “metabolic master switch” regulating pathways of hepatic fat metabolism by phosphorylation modulation of PPAR-α activity [42]. As shown in **Fig.4**, compared with the control group mice, the protein level of phospho-AMPK and the ratio of phospho-AMPK/AMPK were all significantly increased in the liver of ethanol group mice. CMZ co-treatment further increased the protein level of phospho-AMPK.

### CMZ co-treatment increased the phosphorylation of Erk1/2 and p38MAPK

**Figure 5:**
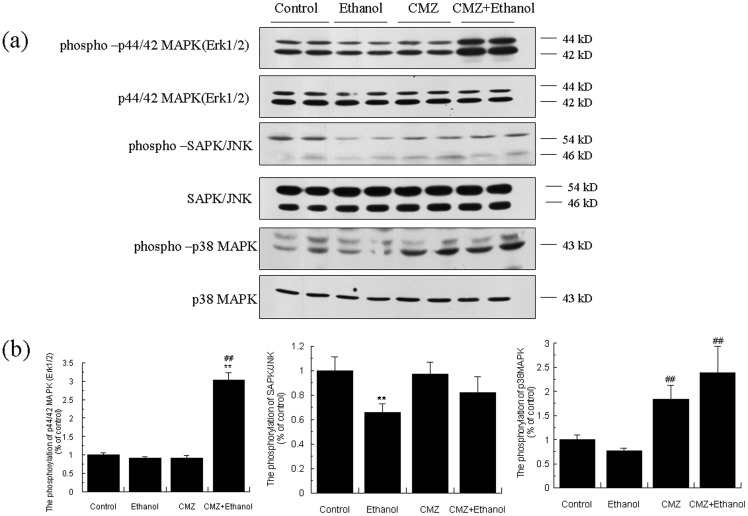
CMZ co-treatment enhanced the phosphorylation of Erk1/2 and p38MAPK. (a) Representative western blot bands for phospho-Erk1/2, Erk1/2, phospho- JNK, JNK, phospho-p38MAPK, and p38MAPK; (b) Quantitative data analyses. Data were presented as mean ± SD from at least 3 independent experiments, and expressed as the percentage of the control. **P<0.01, compared with control group; ^#^ *P<*0.05, ^##^ *P<*0.01, compared with ethanol group.

In addition to AMPK, PPAR-α could be also affected by mitogen-activated protein kinase (MAPK) [42]. Therefore, we investigated the protein levels of total and the phosphorylated form of three kinds of MAPK (Erk1/2, JNK/SAPK, and p38MAPK). As shown in **Fig.5**, the protein levels of phospho-Erk1/2 and phospho-JNK in ethanol group mice liver were significantly decreased when compared with control group mice. CMZ co-treatment significantly increased the phosphorylation of MAPKs. Compared with the ethanol group mice, the ratio of phospho-Erk1/2/Erk1/2 in the liver of CMZ/ethanol group mice was increased to 3.33 fold. Although the phosphorylation of p38 was not significantly affected by ethanol; however, the ratio of phospho-p38/p38 in CMZ/ethanol group mice liver was increased to 3.08 fold compared with that of ethanol group mice. These results suggested that CYP2E1 inhibition by CMZ might lead to the transcriptional activation of PPAR-α.

### CMZ co-treatment led to the activation of PI3K/Akt and the subsequent phosphorylation and inactivation of GSK-3β

**Figure 6:**
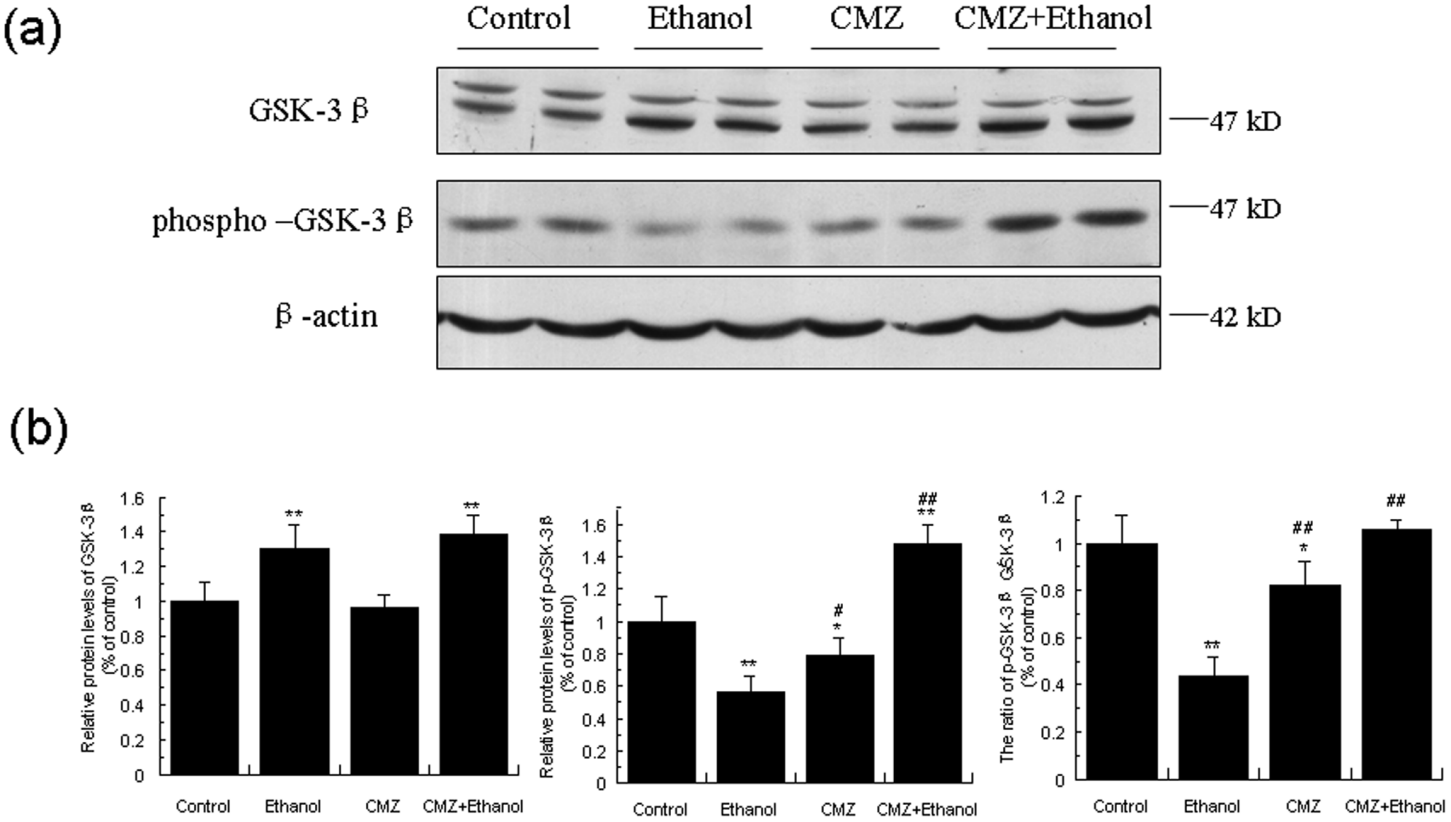
CMZ co-treatment increased protein levels of GSK-3β and phospho-GSK3β in mice liver. Total protein samples were prepared using RIPA buffer, and GSK-3β and phospho-GSK-3β protein levels were detected by western blot. (a) Representative western blot bands; (b) Quantitative data analyses. Data were presented as mean ± SD from at least 3 independent experiments, and expressed as the percentage of the control. *P<0.05, **P<0.01, compared with control group; ^#^ *P<*0.05, ^##^ *P<*0.01, compared with ethanol group.

Glycogen synthase kinase-3β (GSK-3β) is one of the major downstream substrate of PI3K/Akt pathway, and has been demonstrated to modulate the activity of PPAR-α [42], [43]. We then investigated the effects of ethanol and CMZ on several important factors involved in PI3K/Akt/GSK-3β pathway. We firstly investigated the protein levels of GSK-3β, and found that chronic ethanol exposure inhibited the phosphorylation of GSK-3β, shown by the decrease of the phospho-GSK3β^Ser9^ protein level and the increase of total GSK-3β protein level. Interestingly, the phospho-GSK3β^Ser9^ protein levels in CMZ/ethanol group mice liver was significantly increased when compared with the ethanol group mice, which indicated that CMZ co-treatment reversed chronic ethanol-induced decrease of the phosphorylation of GSK-3β (**Fig. 6**).

**Figure 7:**
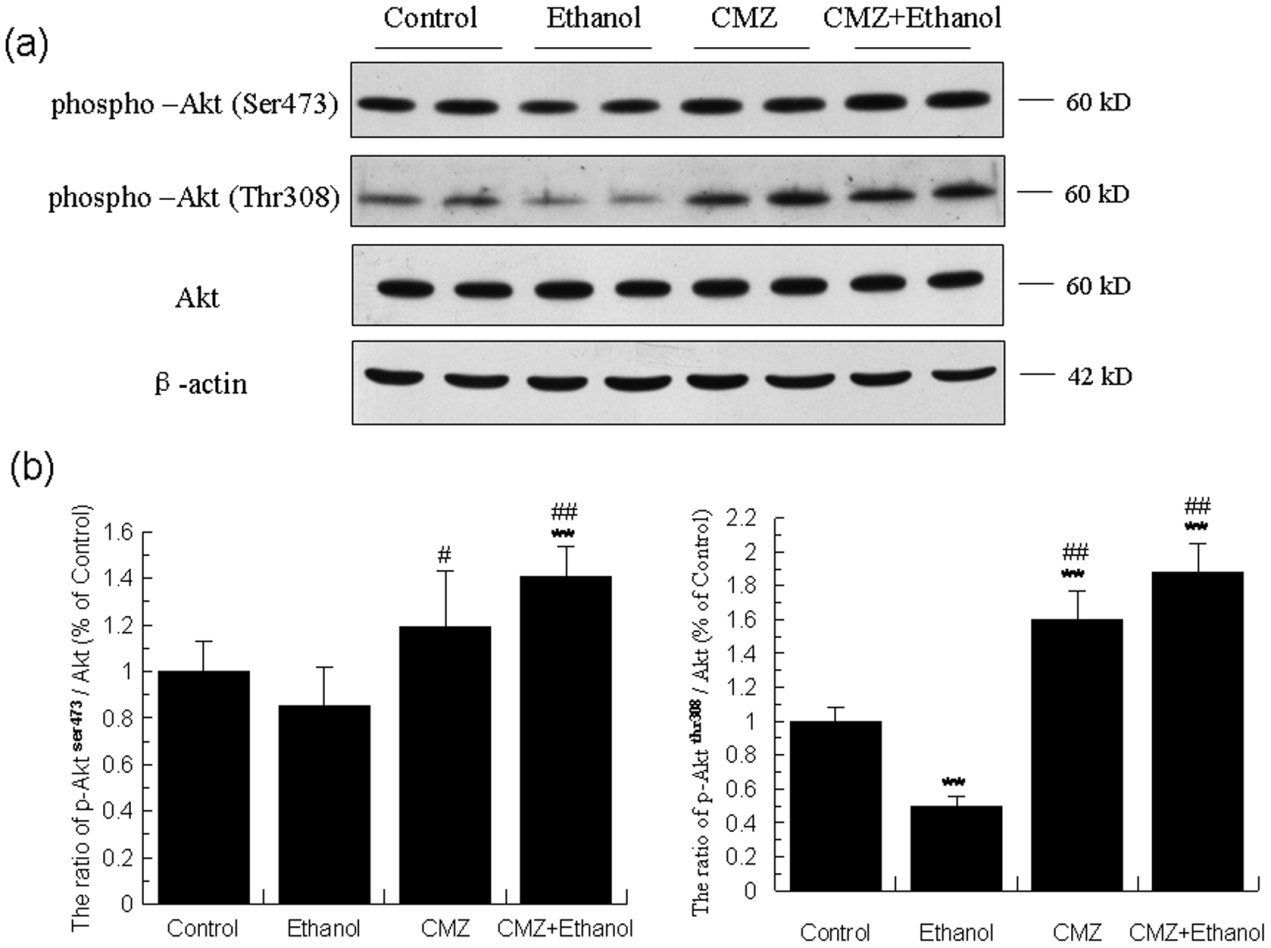
CMZ co-treatment led to the increased phosphoryaliton and activation of Akt. Total protein samples were prepared using RIPA buffer, and protein levels of phospho-Akt^ser473^, phospho-Akt^thr308^, and the total Akt were detected by western blot. (a) Representative western blot band; (b) Quantitative data analyses. Data were presented as mean ± SD from at least 3 independent experiments, and expressed as the percentage of the control. **P<0.01, compared with control group; ^#^ *P<*0.05, ^##^ *P<*0.01, compared with ethanol group.

**Figure 8:**
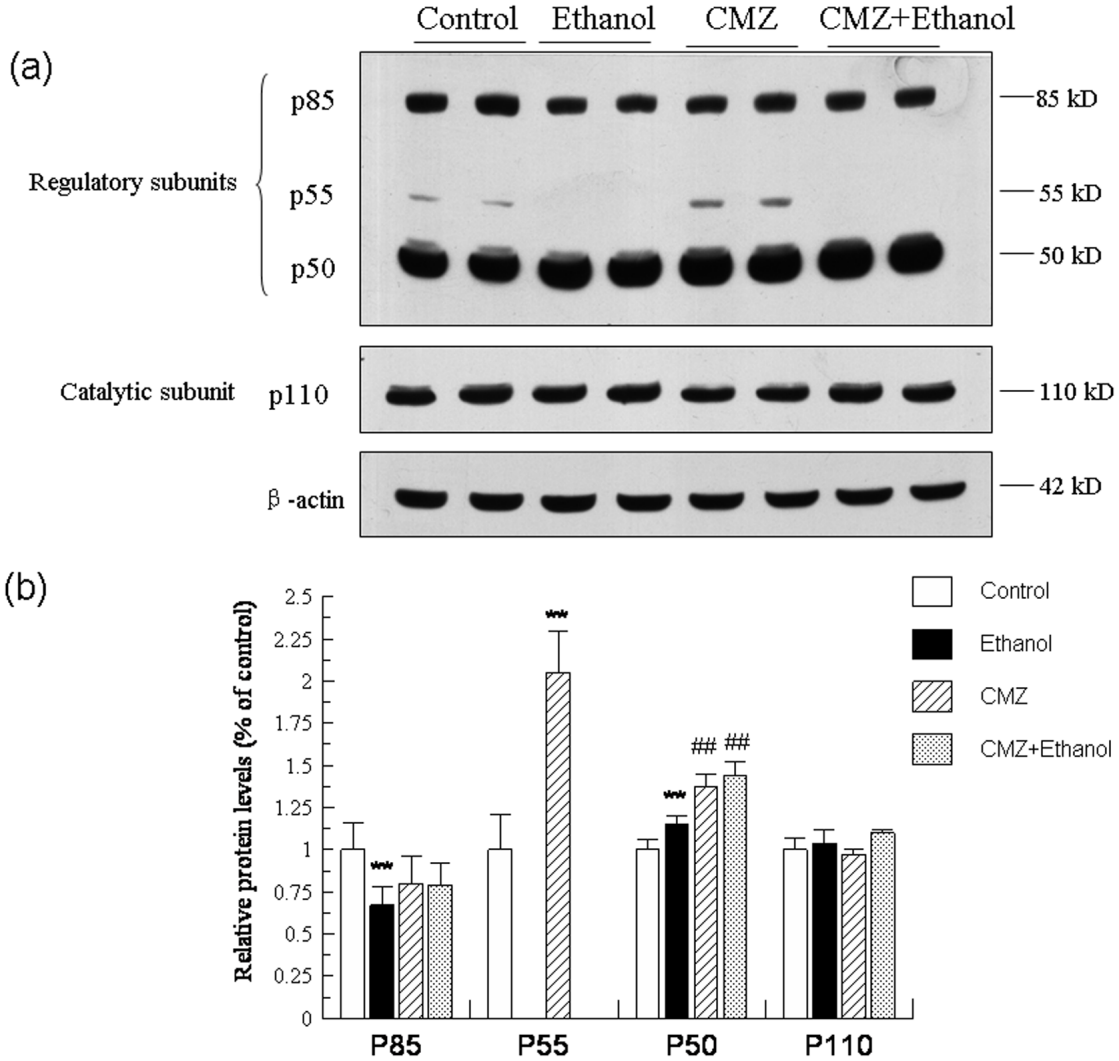
CMZ co-treatment significantly increased the protein levels of p50, the regulatory subunit of PI3K. Total protein samples were prepared by using RIPA buffer, and protein levels of the regulatory subunits of PI3K (p85, p55, p50) and the catalytic subunit (p110) were detected by western blot. (a) Representative western blot bands; (b) Quantitative data analyses. Data were presented as mean ± SD from at least 3 independent experiments, and expressed as the percentage of the control. **P<0.01, compared with control group; ^##^ *P<*0.01, compared with ethanol group.

The changes of Akt were parallel well with that of GSK-3β. As shown in **Fig.7**, the protein levels of total Akt kept unchanged in the liver of different group mice. However, compared with those of control group mice, the protein levels of phospho-Akt^Thr308^ and phospho-Akt^Ser473^ were decreased by 49.73% and 15.50%, respectively. Compared with those of ethanol group mice, the protein levels of phospho-Akt^Thr308^ and phospho-Akt^Ser473^ in the liver of CMZ/ethanol group mice were increased to 3.74 fold and 1.67 fold, respectively. As shown in the **Fig.8**, the protein level of the catalytic subunit of PI3K (p110, 110 kD) was not significantly affected by ethanol and CMZ, while the protein levels of the regulatory subunits of PI3K, p85 (85 kD), p55 (55 kD) and p50 (50 kD), were differently altered in the liver of ethanol group mice. The p85 protein level was decreased by 33.45%, while the p55 was undetectable in the livers of ethanol group mice. Compared with those of ethanol group mice, the p85 protein level in the liver of CMZ/ethanol group mice was increased by 19.26%, while the protein level of p50 was increased by 25.12%. These data suggested that the activation of Akt might be attributed to the up-regulation of the protein levels of the PI3K regulatory subunits, p50.

### CMZ co-treatment reduced the protein levels of mature SREBP-1, blocked the decrease of ACC and FAS, and increased the protein levels of DGAT2

**Figure 9:**
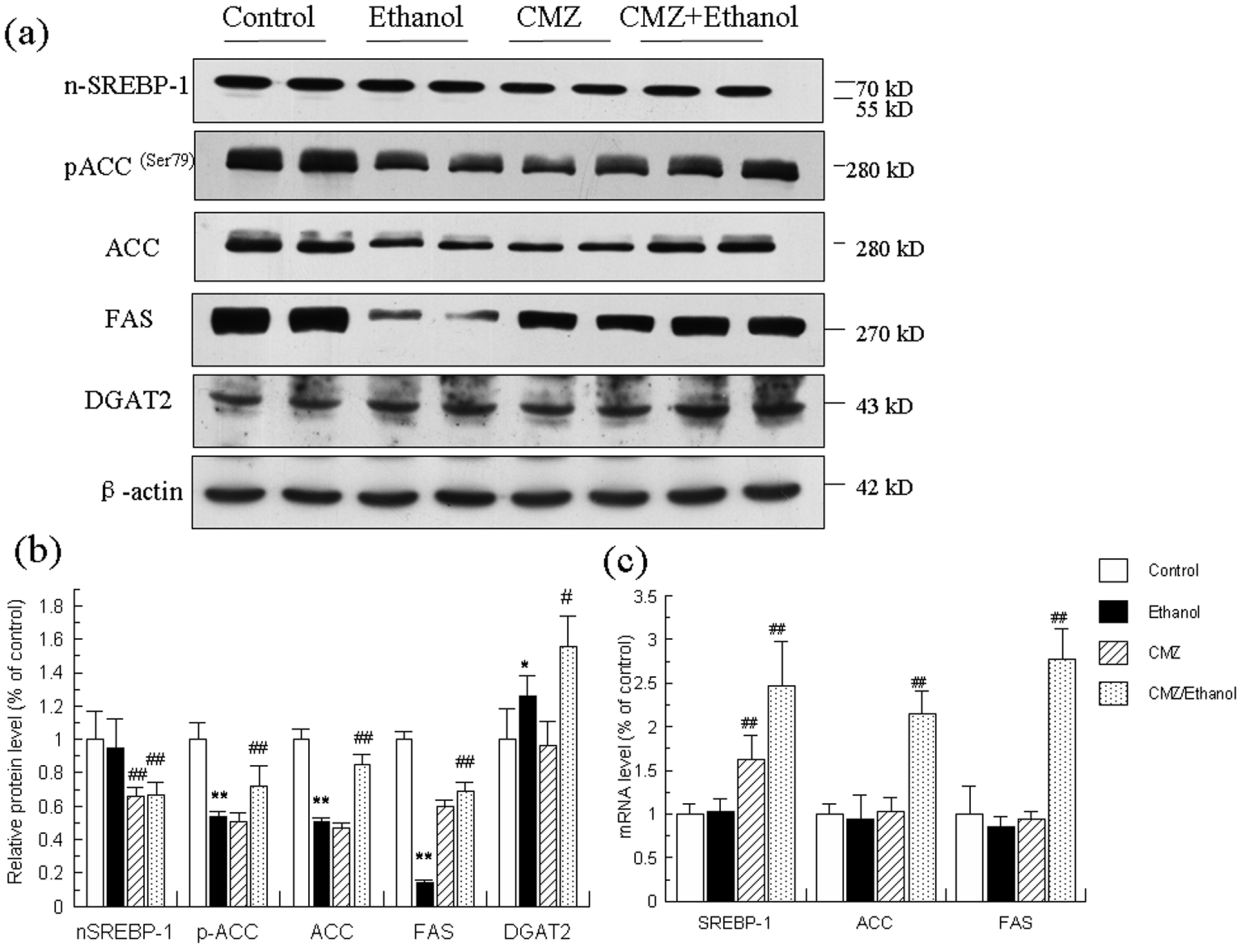
Effects of ethanol and CMZ on the mRNA and protein levels of n-SREBP-1, phospho-ACC^ser79^, ACC, FAS, and DGAT2. (a) Representative western blot bands for n-SREBP-1, phospho-ACC^ser79^, ACC, FAS, and DGAT2. (b) Quantitative data analyses. (c) The mRNA levels of SREBP-1, ACC, and FAS. Data were presented as mean ± SD from at least 3 independent experiments, and expressed as the percentage of the control. *P<0.05, **P<0.01, compared with control group; ^#^ *P<*0.05, ^##^ *P<*0.01, compared with ethanol group.

The mRNA and protein levels of SREBP-1 and two important enzymes involved in fatty acid synthesis (ACC and FAS) were detected and the results were shown in **Fig.9**. We only detected the mature form of SREBP-1 (n-SREBP-1, 68 kD), but not the precursor form of SREBP-1 (125 kD). The mRNA and protein levels of SREBP-1 were not significantly altered in the liver of ethanol group mice when compared with those of control group mice. However, chronic ethanol intake resulted in remarkable decline of the protein levels of ACC, phospho-ACC^ser79^, and FAS, which was significantly suppressed by CMZ co-treatment. The protein level of diacylglycerol acyltransferase 2 (DGAT2), the rate-limiting enzyme in TG synthesis, was significantly increased in ethanol group mice liver, which was further increased in CMZ/ethanol group mice liver.

### Ethanol-activated autophagy might be suppressed by CMZ co-treatment

**Figure 10:**
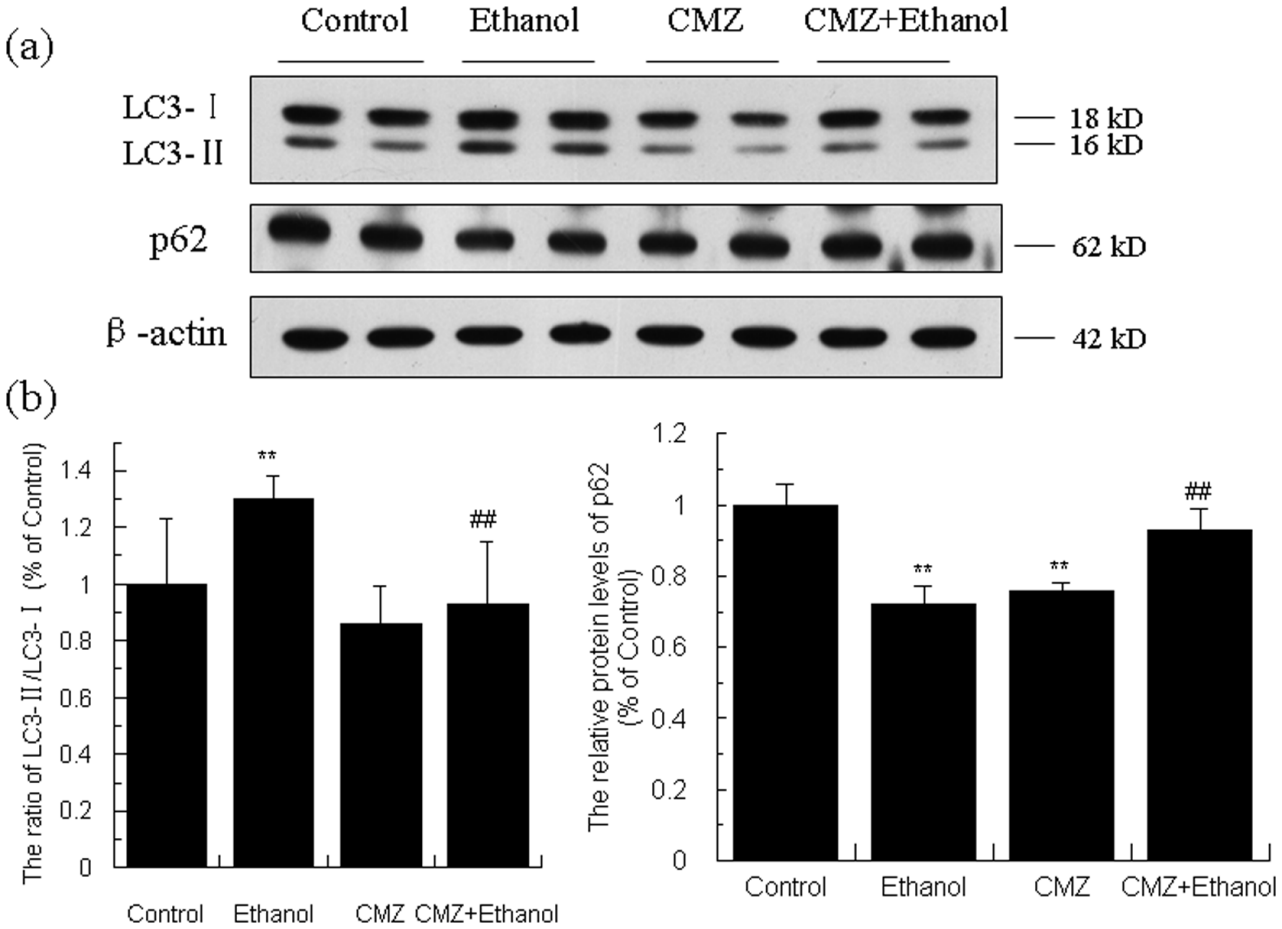
Effects of ethanol and CMZ on the protein levels of LC3 and P62. Total protein samples were prepared using RIPA buffer, and protein levels two biomarkers of autophagy, i.e. LC3 and P62, were detected by western blot. Data were presented as mean ± SD from at least 3 independent experiments, and expressed as the percentage of the control. **P<0.01, compared with control group; ^##^ *P<*0.01, compared with ethanol group.

To investigate whether the CYP2E1 activation could disturb autophagy, we detected the specific biomarkers of autophagy, the ratio of LC3 II/LC3 I and the protein levels of p62. As shown in **Fig.10**, compared with those of the control group mice, the ratio of LC3 II/LC3 I was increased by 57.20% (*P<*0.01), while the protein level of p62 was decreased by 27.97% (*P<*0.01), which indicated that chronic ethanol exposure led to the activation of autophagy. Compared with those of the ethanol group mice, the ratio of LC3 II/LC3 I in CMZ/ethanol group mice was significantly decreased, while the protein level of p62 was significantly increased.

## Discussion

In this study, we evaluated the protective effects of CMZ, a specific CYP2E1 inhibitor, on chronic ethanol-induced fatty liver. The mice were treated with or without CMZ and subjected to 4 weeks of ethanol-containing Lieber-DeCarli liquid diet in which ethanol provided 36% of the energy as previously described [30]. The daily intake of ethanol was about 25-34 g/kg body weight/day. The results showed that CYP2E1 suppression by CMZ completely blocked chronic ethanol-induced fatty liver in mice, which was illustrated by the decrease of the serum and hepatic TG levels and the pathological examination including H&E staining, the specific fat staining (Sudan III and oil red O staining), and the TEM examination (**Table 2** and **Fig. 1**). These results are consistent with some previous studies and support the hypothesis that CYP2E1 plays crucial roles in the development of ethanol-induced fatty liver [15].

To explore the underlying mechanisms for the protective effects of CMZ, we investigated the changes of three fat metabolism pathways, i.e. SREBP-1 mediated lipogenesis, autophagy, and PPAR-α regulated fatty acid decomposition pathway. No significant changes of the protein level of n-SREBP-1c was observed between ethanol group mice and control group mice, while the protein levels of ACC and FAS, the rate limiting enzymes involved in fatty acid synthesis in the liver, were significantly decreased in the liver of ethanol group mice (**Fig. 9**). These results were parallel with the reported decrease in SREBP-1 mediated fatty acid synthesis pathway in some studies [44], [45], but were opposite to others which reported SREBP-1 was activated after chronic ethanol exposure[24], [25]. These inconsistent results might be related with the different composition of the animal diet and the ethanol contents. The mice were treated with low fat liquid diet with less ethanol content (energy provided by ethanol ≤29%) in the later studies, which might affect the expression of SREBP-1c [46]. Chronic ethanol exposure also led to the increase of protein level of DGAT2. CMZ co-treatment significantly suppressed ethanol-induced decrease of ACC and FAS protein levels and further increased DGAT2 protein levels, which indicated that CMZ co-treatment might suppress the decrease of *de novo* fatty acid synthesis and further enhance the TG synthesis. Therefore, the protective effects of CMZ might not be related with the SREBP-1 pathway. Autophagy is a genetically programmed, evolutionarily conserved catabolic process that degrades cellular proteins and damaged and/or excess organelles, which has been suggested to play important roles in ALD[23]. The only reliable methods to reflect the autophagy is to test the autophagy flux, which can be monitored by TEM. However, we did not detect typical autophagosome in the TEM analyses, which might be due to the improper time point of sample collection, as autophagy is a dynamic process and autophagosome is a transient structure. However, the ratio of LC3 II/LC3 I, a wildly used biomarker for autophagy flux, was significantly increased in the liver of ethanol group mice, while the protein level of p62 (another biomarker for autophagy) was significantly decreased, which suggested that chronic ethanol intake led to the activation of autophagy [47], [48]. CMZ co-treatment significantly inhibited the increase of LC3 II/LC3 I ratio and the decline of p62 protein level, which indicated that CMZ co-treatment might suppress the activation of autophagy. These data indicated that the protective effects of CMZ might not be related with the autophagy pathway. These results need to be confirmed in future studies. In regard to the PPAR-α, the mRNA and protein levels were all significantly decreased in ethanol group mice liver, which were consistent with a great number of studies which have demonstrated that PPAR-α suppression play important roles in the pathogenesis of AFL[10], [11], [49], [50]. However, CYP2E1 inhibition by CMZ significantly increased the mRNA and protein levels of PPAR-α (**Fig. 3**). These data suggested that CYP2E1 activation might disturb the activation of PPAR-α, which may contribute to the development of AFL.

CYP2E1 has been reported to be a major contributor to ethanol-induced oxidative stress [37], [38], and oxidative stress has been suggested to play important roles in AFL [51]. It has been reported that ethanol-induced oxidative stress could lead to the overproduction of TNF-α [39], which will then down-regulated the expression of PPAR-α [40]. Therefore, we investigated the changes of MDA, GSH, and TNF-α (**Table 2**). We found that CMZ co-treatment significantly suppressed ethanol-induced increase of hepatic MDA level, and dramatically increased the hepatic GSH level, which suggested that CMZ suppressed chronic ethanol-induced oxidative stress. Furthermore, chronic ethanol exposure-induced significant increase of the serum TNF-α level was completely blocked by CMZ co-treatment. These results suggested that the inhibition of CYP2E1 by CMZ could reduce the oxidative stress and therefore suppressed the overproduction of TNF-α, which might block the decrease of PPAR-α expression.

PPAR-α regulates gene expression by binding with RXR-α as a heterodimer, and then binds to the specific DNA sequence element PPRE. In the absence of ligand, PPAR-α recruits corepressors and histone deacetylases, which reverses histone acetylation, resulting in a more compact chromatin environment in which transcription is repressed [52]. In the presence of ligand for either PPAR-α or RXR-α, the corepressors dissociate so that the ligand can recruit some co-activators including p300 and PGC-1α to activate a series of enzymes involved in fatty acid uptake, activation, and oxidation [36]. We next investigated the changes of RXR-α, its co-activator (p300 and PGC-α), and Sirt-1 (**Fig. 3**). The results showed that RXR-α protein levels was not significantly changed in the livers of mice exposed to ethanol and/or CMZ. However, the protein level of p300 was significantly reduced in ethanol group mice liver, which was partly suppressed by CMZ co-treatment. Although the protein level of PGC-1α was not affected by ethanol or CMZ, the protein level of acetylated PGC-1α was significantly increased in the liver of ethanol group mice, which might be attributed to the decrease of Sirt-1 protein level. Interestingly, CMZ co-treatment completely restored the acetylation of PGC-1α by suppressing the decrease of Sirt-1 protein level. These results suggested that CMZ could suppress the decrease of p300 and Sirt-1 protein levels, and retain the deacetylation of PGC-1α, which might lead to the activation of PPAR-α.

PPAR-α is s phospho-protein and thus its activity could be affected by some protein kinases including AMPK, MAPK, and GSK3 [42]. We then investigated the changes of these protein kinases. AMPK is an energy sensor that is activated when the cell-energy level is low, and has been demonstrated to play important roles in regulating glucose and lipid homeostasis. In contrast to some previous reports in which AMPK phosphorylation was suppressed [45], [53], we noticed that the protein level of phospho-AMPKα^Thr172^ was significantly increased in the liver of ethanol group mice, which indicated the activation of AMPK (**Fig. 4**). These results were not unexpected, as a recently published study has clearly demonstrated that effects of ethanol on AMPK could be influenced by the dietary fat [54]. However, the protein level of phospho-AMPKα^Thr172^ was further increased in CMZ co-treated mice liver. MAPKs are serine/threonine-specific protein kinases, which contain three distinct and parallel MAP kinase cascades including p42/44 MAPK (also called extracellular signal regulated kinases 1 and 2: Erk1/2), p38 MAPK, and c-jun N-terminal kinase or stress activated protein kinases (JNK/SAPK). We recently reported that acute ethanol exposure resulted in significant decrease of the phosphorylation of Erk1/2 and p38MAPK in an acute ethanol-intoxicated mice model [31]. Similar results were observed in the current study, in which the protein level of phospho-Erk1/2 was significantly decreased. However, the phosphorylation of Erk1/2 and p38MAPK was dramatically increased in the liver of CMZ/ethanol group mice (**Fig. 5**). These data suggested that CMZ might activate PPAR-α by activating Erk1/2 and p38MAPK.

In addition to the AMPK and MAPK, another protein kinase, GSK3β, has also been proven to be a regulator of the PPAR-α activity. *In vitro* kinase assays revealed that PPAR-α was a substrate of GSK3β being phosphorylated predominately at serine 73 in the A/B domain, and GSK3β overexpression decreased the stability of PPAR-α, which was abrogated by mutating serine 73 in PPAR-α [42]. In the current study, the chronic ethanol intake led to significant decrease of the phospho-GSK3β^Ser9^ protein level, while CMZ co-treatment resulted in the dramatic increase of the phospho-GSK3β^Ser9^ protein level (**Fig. 6**). As phospho-GSK3β is the inactivated form, it might be speculated that CMZ led to the increased phosphorylation and inactivation of GSK3β, and thus increased the stability of PPAR-α. PI3K and Akt are the upstream kinases of GSK3β [55]. PI3K could convert phosphatidylinositol-4,5-bisphosphate to phosphatidylinositol-3,4,5-trisphosphate (PIP3), which could bind to pleckstrin homology (PH) domain of protein kinase B (PKB/Akt) and phosphoinositide- dependent protein kinase 1 (PDK1), leading to the phosphorylation and activation of Akt, and the phosphorylation and inactivation of GSK3β. We then investigated the protein levels of the upstream PI3K and Akt, and found that CMZ significantly increased the protein levels of phospho-Akt^Thr308^ and phospho-Akt^Ser473^, while chronic ethanol intake impaired the phosphorylation and activation of Akt (**Fig. 7**). In regard to the PI3K, CMZ co-treatment led to a significant increase of the p50 protein level in mice liver (**Fig. 8**). These data indicated that CMZ co-treatment led to the activation of PI3K/Akt pathway, which might phosphorylate and inactivate the following GSK3β.

The study by Lu *et al* has reported that CMZ attenuated hepatic fat accumulation in mice exposed to 2 weeks of Lieber-DeCarli liquid diet [15]. However, that study only reported that CYP2E1 activation might disturb the up-regulation of PPAR-α without further exploration of the underlying mechanisms. The results of the current study explained how CMZ led to the activation of PPAR-α, and also investigated the changes of SREBP-1 pathway and autophagy. However, there is a question that could not be omitted. Although our study and the study by Lu *et al.* demonstrated that CYP2E1 might play important roles in AFL, opposite results have also been reported [16], [17]. Intragastric ethanol infusion model was used in these contradictory studies [16], [17], while voluntary oral ethanol feeding model were used in our study and in that by Lu *et al.* [15]. In the intragastric ethanol infusion model, the mice consumed a little more fat, and the blood ethanol level was much higher than that in our study (200-300 mg/dl vs 190 mg/dl). Therefore, the contradictory results may be associated with the animal model. A direct comparative study using two animal models with same liquid diet contents could explain these contradictory results.

**Figure 11:**
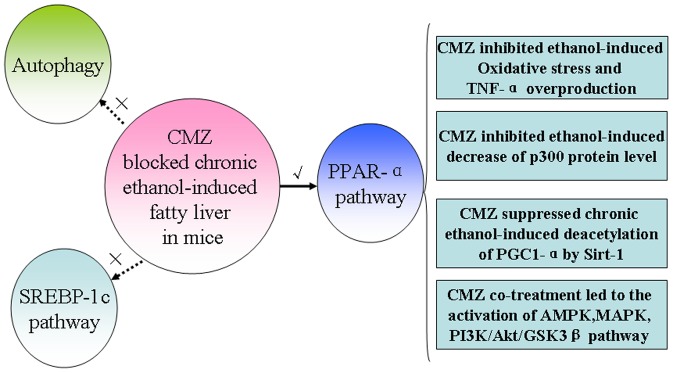
A possible scheme for the protective effects of CMZ against chronic ethanol-induced fatty liver. Ethanol-induced CYP2E1 activation can lead to the suppression of PPAR-α, which may be related with the decline of the p300 protein level, the increase of PGC-1α acetylation, and the disturbance of several protein kinases including AMPK, MAPK, and GSK3β. CYP2E1 activation can also result in oxidative stress, which will lead to the overproduction of TNF-α by activating kupffer cells. In contrast, the protective effects of CMZ against AFL might not be associated with the SREBP-1 mediated lipogenesis and autophagy pathway, which are needed to be confirmed in future studies.

In summary, the current study demonstrated that CYP2E1 suppression by CMZ completely blocked chronic ethanol-induced fatty liver in mice treated with Lieber-DeCarli liquid diet (containing 5% ethanol). CMZ co-treatment significantly inhibited chronic ethanol-induced oxidative stress and the overproduction of TNF-α, and suppressed chronic ethanol-induced decrease of p300 protein level and Sirt-1 mediated deacetylation of PGC-1α. Furthermore, CMZ co-treatment also led to the activation of MAPK (Erk1/2 and p38MAPK) and PI3K/Akt/GSK3β pathway. These factors synergistically led to the stability and the transcriptional activation of PPAR-α, and contributed to the protective effects of CMZ against AFL. In contrast, the SREBP-1 mediated fatty acid synthesis pathway and autophagy associated lipid decomposition might be not involved in the protective effects of CMZ. (**Fig.11**)

## Supporting Information

File S1 **Biochemical data, western blot bands and histological photos used in this study.**
(RAR)
